# Biomechanical properties of predator-induced body armour in the freshwater crustacean *Daphnia*

**DOI:** 10.1038/s41598-017-09649-5

**Published:** 2017-08-29

**Authors:** Sebastian Kruppert, Martin Horstmann, Linda C. Weiss, Ulrich Witzel, Clemens F. Schaber, Stanislav N. Gorb, Ralph Tollrian

**Affiliations:** 1 Department of Animal Ecology, Evolution and Biodiversity; Ruhr-University Bochum, Universitätsstraße 150, 44780 Bochum, Germany; 2 Institute for Product and Service Engineering, Ruhr-University Bochum, Universitätsstraße 150, 44780 Bochum, Germany; 3 Department of Functional Morphology and Biomechanics; Christian-Albrechts-Universität zu Kiel, Am Botanischen Garten 9, 24118 Kiel, Germany

## Abstract

The freshwater crustacean *Daphnia* is known for its ability to develop inducible morphological defences that thwart predators. These defences are developed only in the presence of predators and are realized as morphological shape alterations e.g. ‘neckteeth’ in *D. pulex* and ‘crests’ in *D. longicephala*. Both are discussed to hamper capture, handling or consumption by interfering with the predator’s prey capture devices. Additionally, *D. pulex* and some other daphniids were found to armour-up and develop structural alterations resulting in increased carapace stiffness. We used scanning transmission electron microscopy (STEM) and confocal laser scanning microscopy (CLSM) to identify predator-induced structural and shape alterations. We found species specific structural changes accompanying the known shape alterations. The cuticle becomes highly laminated (i.e. an increased number of layers) in both species during predator exposure. Using nano- and micro-indentation as well as finite element analysis (FEA) we determined both: the structure’s and shape’s contribution to the carapace’s mechanical resistance. From our results we conclude that only structural alterations are responsible for increased carapace stiffness, whereas shape alterations appear to pose handling difficulties during prey capture. Therefore, these defences act independently at different stages during predation.

## Introduction

Predation is a major factor driving the evolution of coexisting species. Prey organisms have evolved different strategies that reduce predation risk. Behavioural adaptations or shifts of life-history parameters mainly reduce chances of predator detection, but may also increase chances of evasion. Morphological adaptations, such as the development of spines or thorns result in changes of overall shape and are interpreted to function when a prey organism is attacked and captured by a predator^1, 2^.

The freshwater crustacean *Daphnia* is known to exhibit a highly plastic reaction to predators and a vast diversity of defensive traits (reviewed in ref. 3). In particular, the variety of morphological adaptations has been the focus of eco-evolutionary research^2, 4–11^ with different species of *Daphnia* able to respond to different vertebrate and invertebrate predators. *D. pulex* De Geer develops ‘neckteeth’ which counter predation by larvae of the phantom midge *Chaoborus* (Diptera)^7^. In contrast, *D. longicephala* develops so called crests in the presence of the heteropteran backswimmer *Notonecta glauca* (Heteroptera; Notonectidae)^9^. For our study we chose these two species due to the different prey-processing strategies of their coexisting predators. The larvae of *Chaoborus* possess a complex feeding basket that transports prey to mouth and pharynx. The modified antennae grasp the prey and pull it towards the mouth. Then the prey is stuffed into the pharynx by alternating mandible movement^12^. *N. glauca* is a visual predator and captures prey using the 1^st^ and 2^nd^ pair of legs. As a heteropteran, *N. glauca* positions its prey to allow penetration with its proboscis followed by injection of proteolytic enzymes and subsequently it imbibes the partially digested tissues^13^.

These inducible morphological defences are discussed to operate as anti-lock-and-key mechanisms, where the morphological trait interferes with the predator’s mouthparts or handling organs and hampers prey consumption^1^. Interestingly, in some species (*D. pulex, D. cucullata* and *D. magna*) these morphological shape alterations are reported to be accompanied by structural changes of the carapace architecture, e.g. thickness and stiffness of the carapace procuticle^14, 15^.

The precise contributions of shape and structural alteration to mechanical resistance against a predation event remain undetermined. In this study, we aimed to analyse the predator-induced changes of overall morphological shape and carapace structure for their effects under the mechanical impact of predator attacks. In addition we wanted to distinguish between the contributions of shape and structure to the carapace’s mechanical resistance and examine whether they work in concert or independently. For this purpose, we measured carapace stiffness using bioindentation at two scales i.e. nanoindentation to assess the procuticle stiffness and microindentation to test the geometric stiffness of the shape. While atomic force microscopy (AFM) is normally used as a surface imaging tool capable of nm scale, it can also be used to conduct nanoindentation measurements at a µN range of forces. We determined the *procuticle* Young’s-moduli (a measure of stiffness) using AFM in ‘contact mode’. With this method we were able to prove whether *D. longicephala* bear an increased carapace stiffness in predator presence. To determine the *structural* Young’s-moduli (the geometric stiffness) of the overall carapace shape, we used micro-indentation. These data were used together with exact morphometric measurements, to create models for physical simulations i.e. finite element analysis (FEA). The morphometric measurements were based on images of carapace structure, conducted with scanning transmission electron microscopy (STEM), and overall morphological shape with confocal laser scanning microscopy (CLSM). With the combination of empiric results and simulations we were able to distinguish explicit aspects of the investigated defences, i.e. the shape and the material structure. For the analysed species we conclude that the procuticle stiffness of the carapace contributes to its increased mechanical resistance. This reduces carapace deformation under force application e.g. during predator capture. The degree of carapace resistance is not, however, affected by morphological shape including neckteeth and crests, indicating that adaptive carapace structure and antipredator morphological shape alterations must act independently rather than synergistically in terms of carapace mechanical resistance. We conclude that the adaptive morphological shape interferes with the predator’s appendages upon capture whereas structural alterations leading to increased carapace resistance counter a predator attack during prey processing.

## Results

### Structure

**Figure 1:**
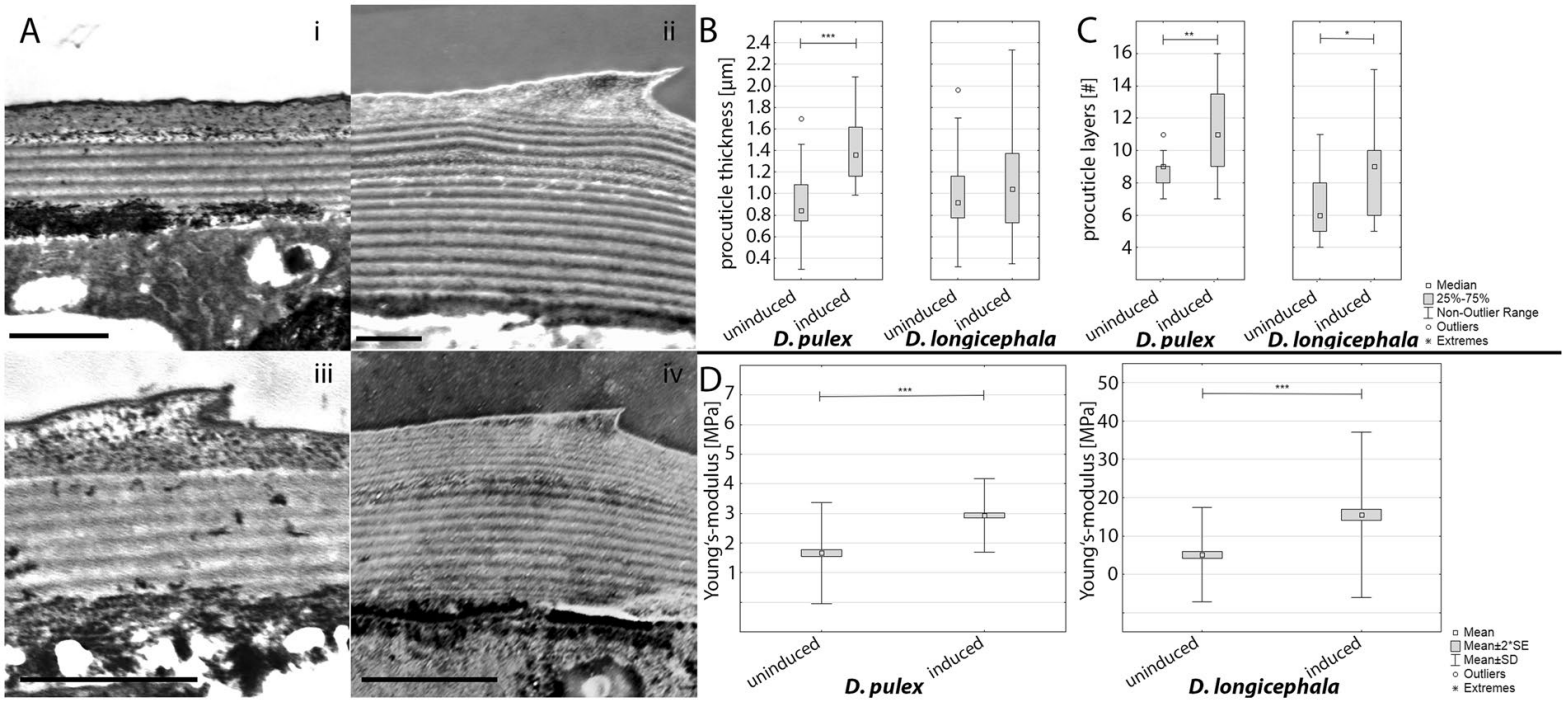
*Procuticle characteristics in* D. pulex *and* D. longicephala. (**A**) STEM images of procuticle cross-sections. (i) *D. pulex* uninduced, scale-bar = 1 µm (ii) *D. pulex* induced, scale-bar = 1 µm (iii) *D. longicephala* uninduced, scale-bar = 1 µm iv) *D. longicephala* induced, scale-bar = 2 µm. (**B**) Procuticle thickness of the uninduced and the induced morphotype of *D. pulex* and *D. longicephala*. (Mann Whitney U-test, *D. pulex:* n (uninduced; induced) = 17, *D. longicephala* n (uninduced; induced) = 20; level of significance: *p ≤ 0.05; **p ≤ 0.01, ***p ≤ 0.005). (**C**) Procuticle number of layers of the uninduced and the induced morphotype of *D. pulex* and *D. longicephala*. (Mann Whitney U-test, *D. pulex:* n (uninduced) = 13, n (induced) = 16; *D. longicephala* n(uninduced) = 21, n(induced) = 19; level of significance: *p ≤ 0.05; **p ≤ 0.01, ***p ≤ 0.005) (**D**) Procuticle Young´s-modulus of *D. pulex (left)* and *D. longicephala* (right) in uninduced and induced form, measured on an AFM. (nested ANOVA, *D. pulex:* n (uninduced; induced) = 5 with min 770 measurements each; *D. longicephala* n (uninduced) = 6, n (induced) = 7 with min 770 measurements each; level of significance: *p ≤ 0.05; **p ≤ 0.01, ***p ≤ 0.005).

Our STEM investigations focused on the distal carapace procuticles of *D. pulex* and *D. longicephala*. In both species the procuticle is organized in chitinous layers (Fig. 1A) with thicknesses of about 1 µm in uninduced morphology. We found significant differences in this organization between the uninduced and induced morphology.

In *D. pulex* the procuticle of the induced morph is significantly thicker (median (uninduced) = 0.843 µm, range = 1.399, median (induced) = 1.358 µm, range = 1.098, Mann-Whitney U-test, U value = 54, p = 0.002; n (induced) = 17; n (uninduced) = 17; Fig. 1B) with a significantly increased number of layers (median (uninduced) = 9, range = 4, median (induced) = 11, range = 9, Mann-Whitney U-test, U value = 43, p = 0.008; n(induced) = 16; n(uninduced) = 13; Fig. 1C).

*D. longicephala* showed no significant differences in procuticle thickness (median (uninduced) = 0.919 µm, range = 1.646, median (induced) = 1.041, range = 1.988, Mann-Whitney U-test, U value = 178, p = 0.56; n (induced; uninduced) = 20; Fig. 1B), but the number of procuticle layers is significantly increased in the induced form (median (uninduced) = 6, range = 7, median (induced) = 9, range = 10, Mann-Whitney U-test, U value = 112.5, p = 0.02; n (induced) = 19, n (uninduced) = 21; Fig. 1C), leading to a decreased layer-thickness in the induced state. The morphotypes of uninduced *D. pulex* and induced *D. longicephala* showed no significant differences in procuticle thickness nor numbers of layers (Mann-Whitney U-test, U value = 143, p = 0.42; n (*D. pulex* uninduced) = 17; n (*D. longicephala* induced) = 20). Thus in the simulations they were represented by the same model.

Material properties for FE analysis were determined by Young’s-modulus measurements of the outer procuticle for induced and uninduced *D. pulex* and *D. longicephala* (Fig. 1D) using AFM. We measured a mean Young’s-modulus of 1.66 MPa ± 1.71 SD for the uninduced morphology and 2.93 MPa ± 1.92 SD for the induced morphology of *D. pulex*. For *D. longicephala* we measured 5.12 MPa ± 12.33 SD in the uninduced morphology and 15.59 MPa ± 21.55 SD in the induced morphology.

**Figure 2:**
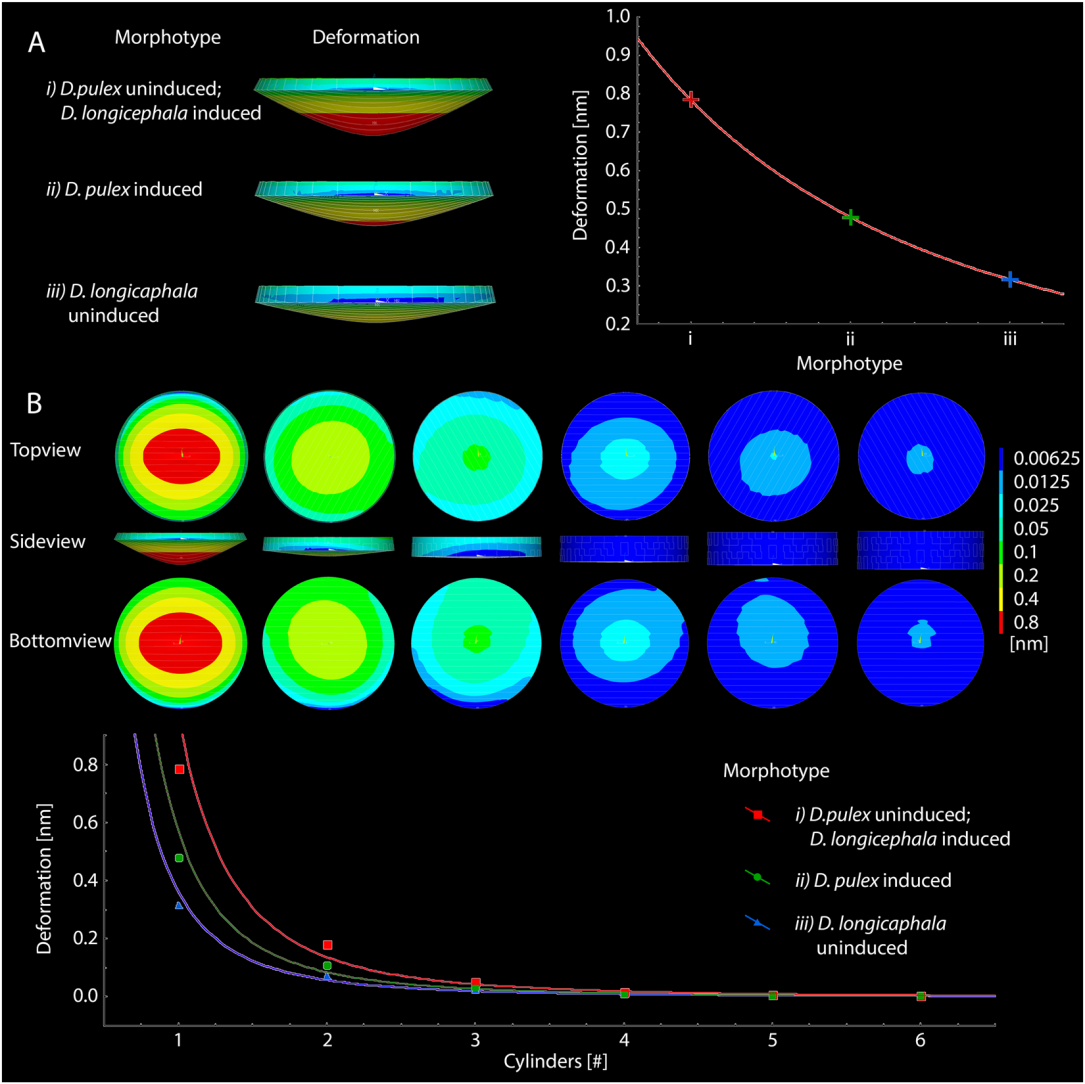
*FEA for the different morphotypes’ procuticle*. (**A**) Simulated deformation of single cylinders used for model-creation of the different morphotypes (Side view); simulated deformation is visually amplified (factor 27). The graph shows the deformation of the single cylinders for the different morphotypes. Curve of best fit: f(x) = 93764x^−2.708^ (R^2^ = 1) (**B**) Simulated deformation of six calculated models with increasing thickness (one to six cylinders), representing morphotype i; simulated deformation is visually amplified (factor 27). The graph shows simulations of maximum deformation at the model’s bottom for all morphotypes. Curves of best fit: i: f(x) = 0.9679x^−2.822^ (R^2 = ^0.9887); ii: f(x) = 0.5703x^−2.745^ (R^2^ = 0.9907); iii: f(x) = 0.3621x^−2.641^ (R^2^ = 0.993).

Based on the observed procuticle layer thickness and number, we created FE models for the different morphotypes and tested their capability to withstand mechanical impact. Simulations of only one cylinder for each morphotype resulted in deformation that decreases with increasing cylinder thickness following a power function (Fig. 2A). The models representing the uninduced morphotype of *D. pulex* and the induced of *D. longicephala* showed the greatest deformation. The procuticle models of uninduced *D. longicephala* showed the weakest maximum deformations at the model’s bottom sides and the models of the induced form of *D. pulex* showed intermediate deformation at model’s bottom side. Comparing the models representing two complete rotations of fibre orientation for uninduced and induced morphotypes within one species, *D. pulex* showed less deformation in the induced morphotype, whereas *D. longicephala* showed less deformation in the uninduced morphotype. Considering the complete set of simulations for the different morphotypes, the deformation at the model’s bottom decreased with an increasing number of cylinders also following power functions (Fig. 2B). Using these curves of best fit to calculate the deformation for procuticle thicknesses observed in STEM, the deformation of *D. pulex* was about 2.4 times higher in the uninduced morphotype than in the induced one. In *D. longicephala* the deformation of the uninduced morphotype was about 1.6 times higher than in the induced morphotype (Table 1).

**Table 1:** Simulated maximum deformation of different procuticle organizations.

**Maximum Deformation at model’s bottom side**			
Number of cylinders in model	*D. pulex* uninduced *D. longicephala* induced	*D. pulex* induced	*D. longicephala* uninduced
1	0.786 nm	0.478 nm	0.316 nm
2	0.182 nm	0.11 nm	0.072 nm
3	0.054 nm	0.033 nm	0.022 nm
4	0.017 nm	0.011 nm	0.008 nm
5	0.009 nm	0.006 nm	0.005 nm
6	0.006 nm	0.004 nm	0.003 nm
**Cylinders to meet procuticle thickness as observed in STEM**	13	16	9
**expected maximum deformation according to fitting**	0.0007 nm	0.0003 nm	0.0011 nm

### Shape

**Figure 3:**
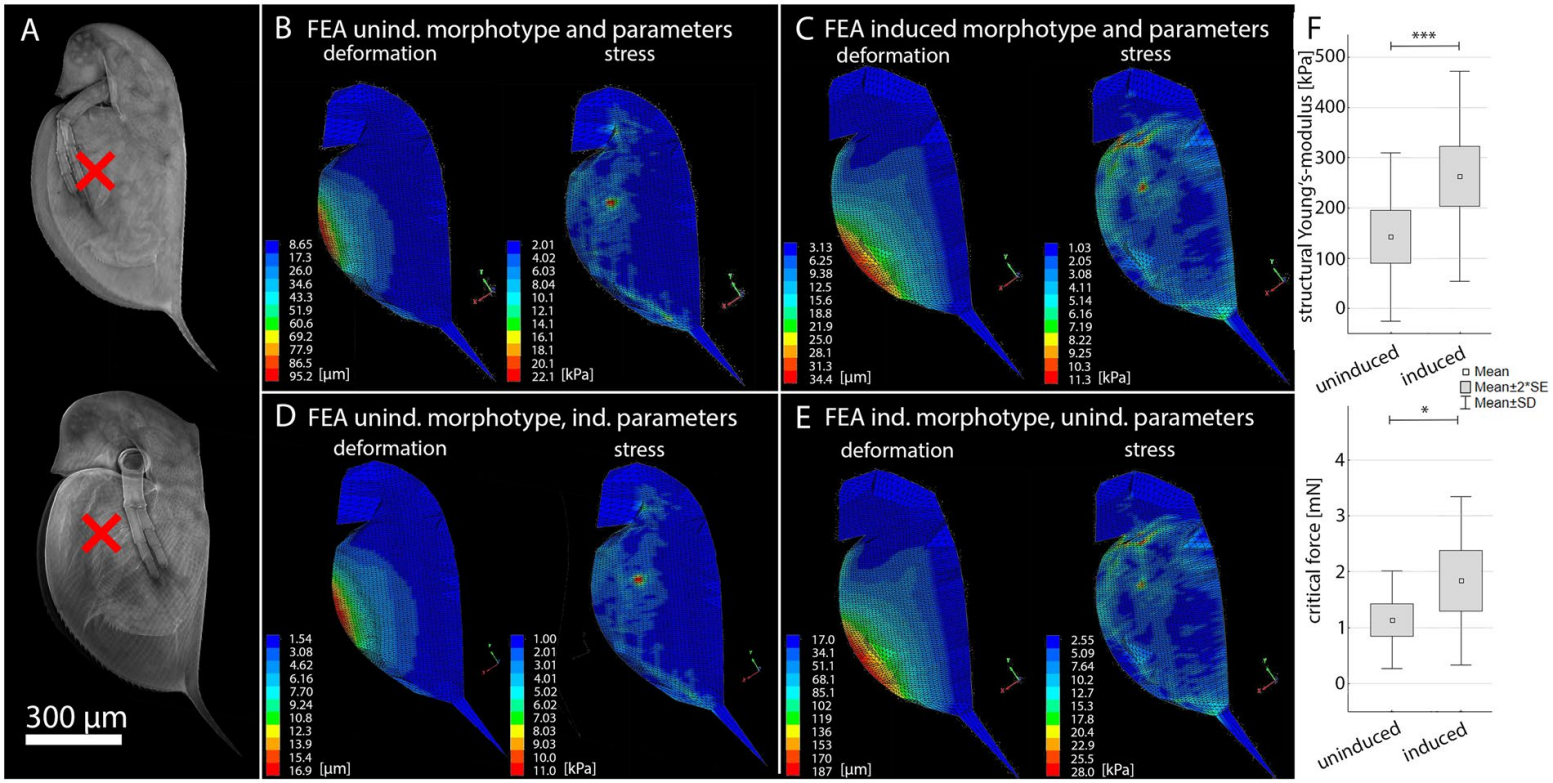
*FEA of D. pulex* (**A**) CLSM projections of uninduced and induced form of *D. pulex*. The red x indicates the region of max lateral width, where the force of 1 mN was loaded onto the models. (**B**) Heat maps of deformation and stress distribution in an uninduced animal’s model. (**C**) Heat maps of deformation and stress distribution in an induced animal’s model. (**D**) Heat maps of deformation and stress distribution in an uninduced model with the material properties of an induced animal. (**E**) Heat maps of deformation and stress distribution in an induced model with material properties of an uninduced animal. (**F**) Empirical data of structural Young’s-modulus and critical force for uninduced and induced animals measured using a microindenter. (t-test, for structural Young’s-modulus measurement n (uninduced) = 41, n (induced) = 50; for critical force measurement n (uninduced) = 36, n (induced) = 31; Level of significance: *p ≤ 0.05; **p ≤ 0.01; ***p ≤ 0.005).

Our CLSM investigations showed the common morphology of *D. pulex* with well-described morphological changes in the induced state i.e. neckteeth in the dorsal head region (Fig. 3A). The FE simulations for the uninduced morphotype, loaded with 1 mN, showed a maximal deformation of 95.2 µm. The deformation was limited to the ventral region of the carapace (Fig. 3B). The stress distribution was not limited to the carapace but also covered the fornices localized in the head region. Maximal observed stress was 22.1 kPa (Fig. 3B). The simulation of the induced morphotype showed a maximal deformation of 34.4 µm and maximal stress of 11.3 kPa (Fig. 3C). The area of deformation included the whole ventral cleft but large deformations were restricted to the posterior half. In comparison to the uninduced model, a wider area of the carapace showed deformation in this simulation. Similarly, the stress distribution showed a larger area of the carapace under high stress. Regions of very high stress were in the area of applied forces and the interceptions of the carapace into the dorsal spine as well as the headshield. Furthermore, the stress in the head region was lower than in the simulation of the uninduced animals.

In a second step we tested shape and structure for their contributions to carapace resistance. This was done by providing a model of the uninduced morphotype, with the procuticle characteristics of induced animals and vice versa. Again we applied a force of 1 mN. The simulation of the uninduced shape with the induced procuticle characteristics resulted in a maximal deformation of 16.9 µm and a maximal stress of 11 kPa (Fig. 3D). The area of deformation and the stress distribution pattern were comparable to the uninduced morphotype. The simulation of the induced shape with the uninduced procuticle characteristics resulted in a maximal deformation of 187 µm and a maximal stress of 28 kPa (Fig. 3E). The area of deformations and pattern of stress distribution was comparable to the simulation for the induced morphotype. Empirical measurements for comparison and validation of the simulations showed an average structural Young’s-modulus of 142.38 kPa ± 167.58 SD in the uninduced and 262.84 kPa ± 209.33 SD in the induced form. We found significant differences between the uninduced and the induced form (*t*-test; *t*-value = −2.98257; *p* = 0.004; Fig. 3F). Additionally, we measured the critical force that results in lethal collapse of the carapace, for both morphotypes. The uninduced morphotype showed an average critical force of 1.14 mN ± 0.87 SD, and the induced 1.84 mN ± 1.50 SD, a significant difference (t-test; t-value = −2.36935; p = 0.02; Fig. 3F). Thus, the micro-indentations showed an increased geometric stiffness and puncture resistance in the induced morphotype.

**Figure 4:**
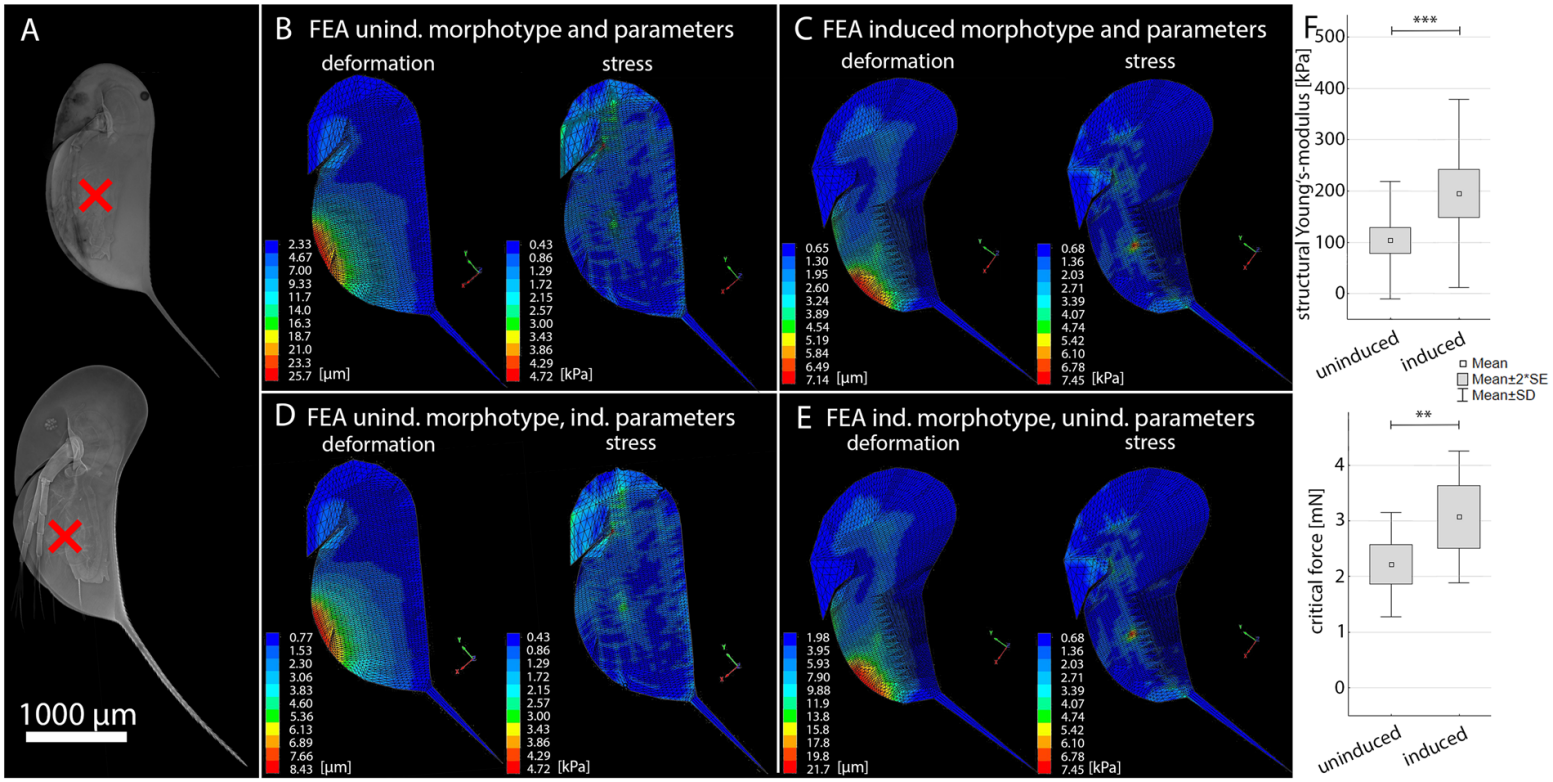
*FEA of D. longicephala* (**A**) CLSM projections of uninduced and induced form of *D. longicephala*. The red x indicates the region of max lateral width, in where the models were loaded for the simulations. (**B**) Heat maps of deformation and stress distribution in an uninduced animal model, loaded with a force of 1 mN. (**C**) Heat maps of deformation and stress distribution in an induced animal model (**D**) Heat maps of deformation and stress distribution in an uninduced model provided with the material properties of an induced animal. (**E**) Heat maps of deformation and stress distribution in an induced animal’s model with the material properties of an uninduced animal. (**F**) Empirical data of structural Young’s-modulus and critical force for uninduced and induced animals measured using a microindenter. (t-test, for structural Young’s-modulus measurement n (uninduced) = 83, n (induced) = 62; for critical force measurement n (uninduced) = 28, n (induced) = 18; Level of significance: *p ≤ 0.05; **p ≤ 0.01; ***p ≤ 0.005).

In *D. longicephala*, we also observed morphological defences: an increase in body and spine lengths, and changes in head morphology, the so-called crest (Fig. 4A). The finite element simulations for the uninduced morphotype, loaded with 1 mN, showed a maximal deformation of 25.7 µm. The deformation was mainly on the ventral region of the carapace but an area on the rostrum also exhibited deformation (Fig. 4B). In comparison to the simulations of *D. pulex*, the stress distribution was not limited to the carapace but included parts of the head, predominantly the fornices. Maximal stress was 4.72 kPa (Fig. 4B). The simulation for the induced morphotype resulted in a maximal deformation of 7.14 µm and a maximal stress of 7.45 kPa (Fig. 4C). The area of deformation was mainly located on the dorsal half of the ventral cleft. In contrast to the simulations of *D. pulex*, the induced morphotype of *D. longicephala* showed a large area of deformation on the head. The stress distribution image showed a smaller area of the carapace under high stress in comparison to the uninduced morphotype. We observed regions of very high stress around the area of the applied force, the head shield and the ventral interceptions of the carapace into the spine. In the induced morphotype, the stress in the head region was lower than in the simulation of the uninduced animals.

We also tested whether the form and structure of the carapace act in concert or independently. The simulation of the uninduced morphotype with procuticle characteristics of induced animals showed a maximum deformation of 8.43 µm and maximum stress of 4.72 kPa (Fig. 4D). The area of deformations and pattern of stress distribution was the same as in the simulation with the uninduced morphotype and only differed in magnitude of deformation. The simulation of the induced morphotype with the uninduced procuticle characteristics resulted in a maximal deformation of 21.7 µm and a maximal stress of 7.45 kPa (Fig. 4E). The area of deformation and the stress distribution pattern were comparable to the induced morphotype. In *D. longicephala* we measured a structural Young’s-modulus of 103.96 kPa ± 114.34 SD for the uninduced and 195.39 kPa ± 182.98 SD for the induced morphotype (Fig. 4F). As for *D. pulex*, the *t*-test showed significant differences between the uninduced and the induced form (*t*-value = −3.69050; p = 0.0003). We determined a critical force of 2.22 mN ± 0.94 SD for the uninduced and 3.07 mN ± 1.18 SD for the induced morphotype (*t*-test; *t*-value = −2.71480; p = 0.009; Fig. 4F). Thus, the micro-indentations showed an increased geometric stiffness and puncture resistance in the induced morphotype.

## Discussion

Daphniids are prominent for their inducible morphological defences. Recently it has been shown that besides the distinct shape alterations, so called hidden defences exist within the carapace structure that result in a higher carapace resistance^14, 15^. We developed a method to identify structural features of the carapace architecture responsible for the stiffness increase observed in *D. pulex* and *D. longicephala*. Although alterations in daphniids’ carapace stiffness have been reported before for *D. pulex*, *D. cucullata*, *D. middendorffiana* and *D. magna* ^15–17^ the underlying mechanism has not been described yet. Furthermore, we present a method to distinguish between the contributions of structure and shape to the mechanical resistance of the carapace. FEA is a tool with the ability to analyse single elements of complex biological defence structures, particularly in small invertebrates where empiric data acquisition is limited. We used FEA to determine the physical features contributing to the protective effect of morphological alterations. Our experimental data show increased carapace stiffness in the induced morphotype of both analysed species that are characterized by different inducible morphological traits.

### Structure

Predator-induced changes in procuticle structure result in an increase of procuticle thickness and number of layers in *D. pulex* but a constant thickness accompanied by an increased number in layers in *D. longicephala*. Both induced forms show a significant increase in the procuticle Young’s-moduli in our experiments and thus higher carapace stiffness. Although the AFM data revealed high inter- and intra-individual variance in the measurements, the data was normally distributed and the nested ANOVA showed significant differences between induced and uninduced animals for both species.

We simulated the different procuticle features of the different morphotypes using FEA. Our results indicate that the induced morphotype of *D. pulex* realizes the increase in carapace stiffness by higher procuticle thickness, as well as a higher number of layers. This type of defence seems to be a combination of two beneficial effects where an increase of puncture resistance due to a higher level of lamination is combined with an increase of flexural strength due to higher thickness. Chitin fibres are strongest against tension in their fibre direction and therefore a compound material like the procuticle is strongest if there is a fibre orientation matching every possible loading direction. A higher level of procuticle lamination equates to a higher number of continuous rotations of fibre orientation and thus more fibres for every possible loading direction. The resulting increased puncture resistance in *D. pulex* is accompanied by increased flexural strength i.e. crush resistance since flexural strength increases with material thickness. *Chaoborus* larvae grasp their prey with a rapid movement and ingest it by alternating mandible movement. During this handling or even during ingestion an increase in flexural strength may be beneficial by reducing damage caused by head appendages or mandibles. While daphniids in early instars are unlikely pierced or crushed during ingestion because they easily fit into the mouth, later instars may benefit from prior increased flexural strength when forced through the mouth opening. However, the increased flexural strength may be beneficial even for early *D. pulex* instars when facing earlier instars or smaller species of *Chaoborus* for comparable reasons. In conclusion, the structural alterations might increase *D. pulex*’s survival chances during predator handling. Thickening of protective structures as a reaction to predator presence/threat is known from different invertebrate species e.g. the mussel *Mytilus edulis*, the snail *Littorina*, and Odonata larvae^18–20^ and is repeatedly discussed as an inducible defence.

In *D. longicephala* the induced morphotype displays an increase in procuticle layers at a constant procuticle thickness in comparison to the uninduced morphotype. This indicates an increase in carapace puncture resistance only. *D. longicephala* counters predation of a heteropteran predator that punctures the carapace with its proboscis. Thereby, the likelihood of successful penetration may be reduced through this procuticle rearrangement. We thus anticipate that *D. longicephala*’s procuticle acts like a bullet proof vest against *Notonecta*’s proboscis. Similarly, it is known from mantis shrimps (Stomatopoda) that their telson is optimized in lamination pattern, thickness and mineralization to withstand opponent’s strikes in ritualized fights^21, 22^.

The structural alterations in both analysed species indicate a defensive effect that specifically counters consumption of the coexisting predator.

We have shown that procuticle reorganization has a crucial impact on carapace resistance but other effects may contribute too, e.g. chemical composition, fibre crosslinking or mineralization^23–26^. Since the crustacean cuticle is a very versatile structure with numerous parameters defining the overall properties, it is possible that chemical analysis of the carapace composition could reveal additional strategies for enhancement of its defensiveness.

### Shape

We aimed to determine whether the adaptive defence morphologies of *Daphnia* actually affect overall deformation during punctual force application. We modelled the *D. pulex* and *D. longicephala* uninduced and induced overall shape using a classical landmark-based system and combined these with the measured procuticle Young’s-moduli and thicknesses. The extracted models of both species matched both shape outlines and thus reflected natural appearance. The simulations in which we applied the naturally relevant force of 1 mN resulted in decreased deformation in the induced morphology compared to the uninduced for both species. In *D. pulex* this was a 64% reduced deformation of the induced morphology in comparison to the uninduced. The area of deformation as well as the distribution of stress was greater in the induced morphology model, which might explain overall reduction of stress (~50%) and deformation. This is supported by the experimentally determined Young’s-modulus, which increased by 85% and the critical force for lethal injury by 62%.

The simulations of *D. longicephala* resulted in a 72% reduced deformation of the induced morphology model in comparison to the uninduced model. In contrast to *D. pulex* the induced morphology possesses large modifications (i.e. the crest) but the thickness of the procuticle is the same as in the uninduced morphology. In the uninduced model, the greatest deformation was found at the ventral cleft positioned half the distance from head to tail spine. The induced morphotype’s maximum deformation was also positioned at the ventral cleft but positioned in the lower third of the head to tail spine distance. The stress distribution was more concentrated but showed higher maxima within the area of impact. Therefore, the induced morphology reduced the deformation significantly but revealed higher local stress maxima. Our experimental data of the carapace structural Young’s-modulus confirms this increase of carapace resistance. We found a Young’s-modulus increase of 88% and the critical force showed an increase of force necessary to provoke lethal injury of 72%.

To test whether the protective effect originates from the overall shape or the underlying structure we simulated the induced shape together with the procuticle Young’s-modulus and structure of an uninduced animal and vice versa. In *D. pulex* the induced shape combined with the uninduced structure showed a deformation nearly twice as high as the uninduced morphology. The simulation of the uninduced shape combined with the induced structure resulted in a deformation only half the magnitude of the induced morphology. Our results indicate that the shape alteration actually negatively influences the carapace resistance in *D. pulex*, which is compensated by changes of the procuticle structure. In *D. longicephala* we observed a marginally reduced deformation in the simulation of the induced shape combined with the uninduced structure in comparison to the uninduced morphology. The uninduced shape combined with the induced structure showed deformation almost identical to the induced morphology. In contrast to *D. pulex* the shape of *D. longicephala* has no negative effect on the carapace resistance, but the increase in carapace resistance is mainly based on the structural alterations. The lacking contribution of shape alterations to the carapace resistance in both species indicates that the defensive effect of these alterations is rather to impede predator handling or capture as was hypothesized before^7, 11^.

## Conclusion

Overall, our results indicate that mechanical resistance originates from the structural reorganization of the procuticle rather than the overall shape. Nevertheless, one factor explaining the evolution of inducible defences is that such traits are beneficial, consequently neckteeth and crests must incur a different protective effect^3^. We anticipate that defensive morphological traits will pose handling difficulties on the predator and therefore act during a different portion of a predation event i.e. during capture and supports the anti-lock-and-key hypothesis. Likewise, morphology-dependent hydrodynamic advantages may improve swimming performance and reduce capture risk. In contrast, increased mechanical resistance resulting from procuticle reorganization may act during predator handling or ingestion, so that the probability of prey ingestion (in the case of *Chaoborus*) or successful proboscis penetration (in the case of *Notonecta*) is reduced.

## Materials and Methods

### Experimental organisms

Age-synchronized *Daphnia pulex*, clone R9 (originating from Canada) and *Daphnia longicephala*, clone LP1 (from Lara Pond, Australia) were cultured under constant conditions with a day:night cycle of 16:8 hours at 20 °C ± 0.1 °C in a climate cabinet. Both species were cultured in 1 L glass beakers (WECK®; Germany) containing charcoal-filtered tap water and were fed *ad libitum* with the algae *Scenedesmus obliquus*. Population density was kept below 30 individuals per 1 L to ensure stable population growth. *Chaoborus obscuripes* and *Notonecta glauca* were captured in ponds of the botanical garden of the Ruhr-University Bochum, Germany. *C. obscuripes* was kept at densities of 50 individuals in 1.5 L beakers at 4 °C ± 1 °C. *N. glauca* was kept at densities of 5 individuals in 10 L buckets at 20 °C ± 1 °C. Both predators were fed regularly with daphniids every 48 hours.

### Induction of morphological defences in *D. pulex* and *D. longicephala*

Induction of morphological defences was performed in triplicate, by exposing *D. pulex* to chemical cues released from actively feeding *Chaoborus* larvae (*C. obscuripes*). We transferred ten 4^th^ instar *Chaoborus* into a net-cage (mesh size 100 µm) placed in 1 L beakers filled with charcoal-filtered tap water. These larvae were fed with 100 juvenile *D. pulex*. The net-cage prevented encounter between the predator and the test animals but allowed exchange of biologically active chemical cues. Test animals were maintained outside the net-cage and fed *ad libitum* with the green algae *S. obliquus*. We introduced 10 daphniids carrying black-eyed embryos in their brood pouches. Once the neonates were released the mothers were removed. When the offspring moulted into the second juvenile instar, they were collected and analysed. Controls were performed likewise, but in the absence of the predator.

Morphological defences in *D. longicephala* were induced similarly, but using *N. glauca* as predator. We transferred 25 first instar *D. longicephala* into 1 L of charcoal-filtered tap water. *Notonecta* was fed with 10 additional adult *D. longicephala* in the net-cage. *S. obliquus* served as food for *D. longicephala*. Predator and prey were fed every 48 h. Once neonates had reached maturity, they were analysed.

### Procuticle analysis

We used STEM imaging to analyse the *Daphnia* procuticle. Specimens were fixed in 1% glutaraldehyde (VWR, Radnor, USA) diluted in phosphate buffered saline (PBS, 0.1 M, pH 7.4) overnight and rinsed in PBS 3 times for 30 min. We contrasted them with 2% osmiumtetroxyde solution (Heraeus, Hanau, Germany) diluted in PBS (0.1 M, pH 7.4) for 40 min and subsequently rinsed in deionized water 3 times for 30 min. Dehydration was performed with an ascending ethanol series: 15 min in 50% EtOH, 8 h in 70% EtOH, 25 min in 90% EtOH, 5 min in 100% EtOH and finally 2 times 30 min in 100% EtOH. Infiltration with Agar 100 (Agar Scientific, Essex, United Kingdom) was carried out according to the manufacturer’s protocol; in brief: 2 h in 33% Agar 100 medium diluted in EtOH, 2 h 66% Agar 100 medium diluted in EtOH, 2 h in 100% Agar 100 medium. Subsequently, the samples were transferred into Teflon moulds (Sigma-Aldrich, Chemie GmbH, Munich, Germany) filled with 100% Agar. Polymerisation was performed at 60 °C for 48 h. The embedded specimens were cut (45–70 nm thickness) using an ultra-microtome (Reichert Jung Ultracut E, Leica Microsystems, Wetzlar, Germany) equipped with a diamond knife with a boat and a 2.5 mm edge (Diatome 45°, Diatome, Hatfield, PA, USA); the knife angle was set to 7°. The floating sections were expanded using xylol fumes diffusing from a wooden stick. Afterwards the sections were directly transferred to sample-grids (Stork Veco B.V. Eerbeek, Holland). We used copper TEM grids with a mesh width ranging from 20 to 80 lines per cm. STEM imaging was conducted on a Zeiss Gemini (Zeiss Gemini Sigma VP, Zeiss, Oberkochen, Germany) with the acceleration voltage set to 20 kV and the detector set to ‘dark field segment mode’. The acquired images were analysed for procuticle thickness using the software Zeiss SmartTiff and the number of layers was counted (Version V02.01, Carl Zeiss Microscopy Limited, Cambridge, United Kingdom).

### Procuticle Young’s-modulus

**Figure 5:**
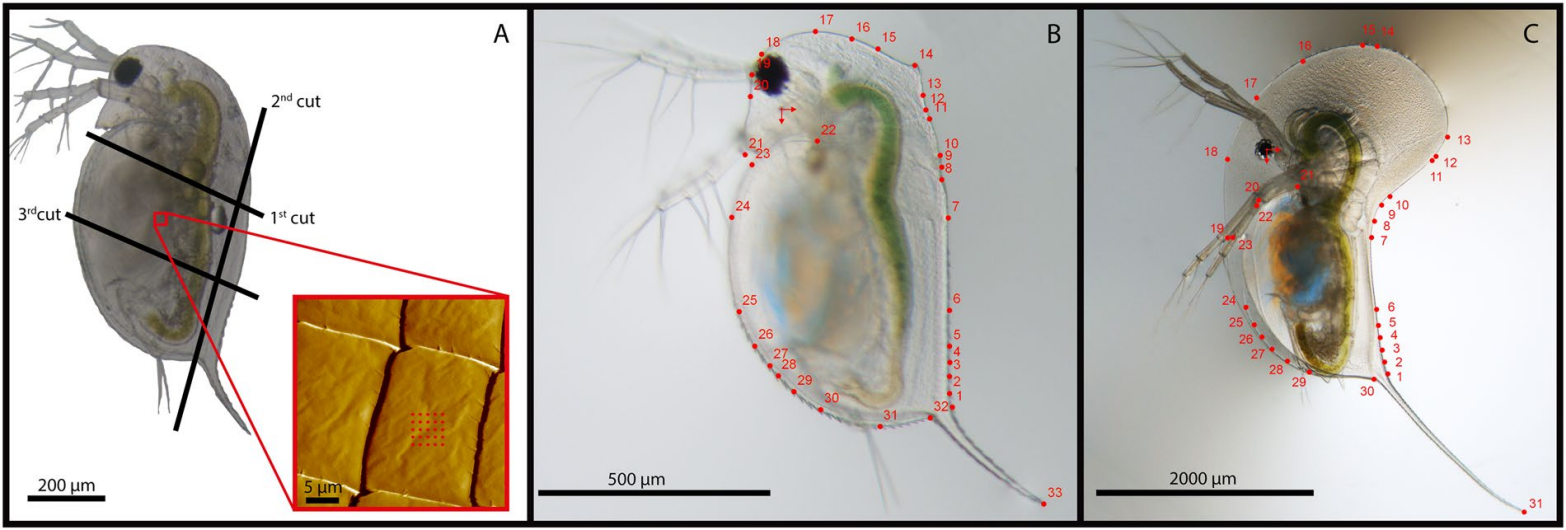
Sample preparation and data uptake for different measurements. (**A**) Scheme of preparation and measurement of daphniids on the AFM. Lines indicate the position and sequence of sections tending to create flat carapace sections. Insert shows an AFM surface scan of the created sections, revealing the shingle like pattern, red dots representing an indentation measurement matrix. **Β:** Defended *D. pulex* in 2^nd^ juvenile instar. The numbers indicate the landmarks for the shape reconstruction referring to Table 1. Scale bar = 500 µm. (**C**) Defended sexually mature *D. longicephala*. The numbers indicate the landmarks for the shape reconstruction referring to Table 1. Scale bar = 2000 µm.

To measure the procuticle Young’s-modulus we applied AFM. For this purpose, we randomly selected induced and uninduced individuals from both species. The carapace of each individual was dissected and cut with a razor blade into two or three rectangular samples of approximately 500 µm edge length (*D. longicephala*) or approximately 200 µm edge length (*D. pulex*) (Fig. 5A). The samples were mounted on an object slide (Menzel GmbH & Co KG, Braunschweig, Germany) coated with a fine layer of Vaseline (Elida Fabergé, Hamburg, Germany), and transferred into a Petri dish filled with charcoal-filtered tap water. A randomly chosen area (30 µm^2^) of the sample was scanned using an Atomic Force Microscope (NanoWizard, JPK Instrument AG, Berlin, Germany) in phase contact mode. Based on the acquired image, measurement spots for the indentation were determined within the flat areas of the shingle structures defining the carapace’s surface pattern (Fig. 5A). Up to three sets of indentation measurements were performed per single shingle and up to three shingles were used for measurements in each area scanned. Measurements were conducted by defining a region of interest as a square of 5 µm. In total 25 indentation measurements were performed in a micrometre distance within the region of interest (Fig. 5A). In total, we analysed 5 induced and 5 uninduced *D. pulex* and 7 induced and 6 uninduced *D. longicephala*. Due to this multi-point data acquisition strategy, we performed a nested ANOVA to test for significant differences between induced and uninduced morphotypes. Maximal indentation force was set to 10 nN and maximal indentation depth to 50 nm. For the measurements a silicon nitride cantilever with a square based pyramid tip (ORC8-10, Bruker Corporation, Billerica, MA, USA) with a nominal tip radius of 15 nm and a spring constant of 0.1 N/m was used. The cantilever properties were checked regularly with test measurements on a glass Petri dish: the spring constant ranged from 0.08 to 0.12 N/m; the tip radius was 33 nm. The Young’s-modulus for each measured point was calculated from the resulting force–distance curves using the manufacturer’s software (JPK DP, JPK Instrument AG, Berlin, Germany). The calculations were based on the Hertz model for four-sided pyramidal indenters:

1 $$E=\frac{F\times \tan \alpha }{1-{\vartheta }^{2}\,\sqrt{2}}\,{\delta }^{2}$$

*E = *Young’s-modulus

*F = *force

*α = *cantilever face angle

*δ = *indentation depth

$$\vartheta $$ = Poisson’s ratio

**Equation** (1): Hertz model for four-sided pyramidal tips

### Carapace structure and finite element analysis

Based on the collected data on carapace structure and stiffness, we modelled the material behaviour upon mechanical impact *in silico*. We performed FEA with the help of the software ANSYS (version R15.0, ANSYS Inc., Canonsburg, PA, USA). We analysed the patterning of the procuticular layers observed in the STEM cross-section images whose grey scale continuously alternates from bright to dark representing the horizontally rotating orientation of chitin fibres. Every bright–dark stripe of the procuticle represents a lamella with fibre rotation in successive monolayers of fibres between 0° and 180° and was defined as one layer^27^. This complete rotation cannot be modelled using FEA since the fibre rotation through the procuticle is virtually continuous. Therefore, we chose to simulate the procuticle in a stepwise approach by successively modelling the procuticle structure in steps of 120° fibre rotation. Furthermore, a model for the complete procuticle thickness exceeds soft- and hardware limitations. Hence we simulated at least a fibre rotation of 720° in total. We started with the simulation of one cylinder, representing a procuticle fraction with fibre rotation of 120°. Subsequently cylinders with an offset of 120° were added up to a total number of six cylinders to simulate a 720° fibre rotation (see Supplementary Fig. S1). Based on these simulations we calculated a curve of best fit for deformation dependent on the number of cylinders. This curve was finally used to extrapolate the deformation for the complete procuticle thickness observed in STEM.

For model creation the individual cylinder thickness was adjusted to the measured procuticle thickness by determining the mean thickness of 120° fibre rotation within the procuticle defined as:

2 $${{\rm{T}}}_{{120}^{^\circ }}=\frac{{{\rm{T}}}_{{360}^{^\circ }}}{3}$$

**Equation** (2): Thickness of a cuticle section representing 120° fibre rotation

While T_360°_ was:

3 $${{\rm{T}}}_{{360}^{^\circ }}=\frac{{\rm{procuticle}}\,\mathrm{thickness}\,[\mu {\rm{m}}]}{{\rm{procuticle}}\,\mathrm{layers}\,[\#]}\times 2$$

**Equation** (3): Thickness of a cuticle section representing 360° fibre rotation

Cylinder diameter was set to 1.5 µm due to model size limitations. To simulate the chitin-protein matrix within the procuticle, we applied two different material-properties for the cylinders. Therefore, the cylinders were subdivided vertically with alternating material properties. Chitin fibres are represented in our model with a Young’s-modulus of 17 GPa and a Poisson’s ratio of 0.3, while the protein matrix, in which the fibres are embedded, was assigned a Young’s-modulus of 0.2 GPa and a Poisson’s ratio of 0.3. The chosen Young’s-modulus of 17 GPa lies within the range of single cellulose fibres^28^, which are chemically comparable to chitin and the Poisson’s ratio of 0.3 is typical for compound materials. The matrix Young’s-modulus was set to a typical dimension for compound materials in which the Young’s-modulus of the implemented fibres is distinctly higher than that of the matrix.

Conditions for all simulations were as follows: all edge-nodes of the bottom surface were blocked for movement in the z direction, one of these nodes lying on the y-axis was additionally blocked for movement in the x direction and the node lying on the opposite side of the y-axis was blocked for movement in all directions. This represents a standard practice for loading simulations with one direction of interest. A force was applied on five nodes (in sum: 3 mN) in the centre of the respective top surface area.

This workflow was conducted for induced and uninduced morphotypes of both analysed species.

### Optical imaging of carapace shape

To measure the body outline of the induced and uninduced morphotypes of both species, they were transferred onto a glass slide. All images were taken on a stereo microscope (Olympus SZX 16, Olympus, Hamburg, Germany) with mounted digital camera (Colorview III, Soft imaging Systems, Hamburg, Germany) and the software Cell^D (Olympus, Hamburg, Germany). To create representative datasets, 11 uninduced and 12 induced *D. pulex* as well as 13 uninduced and 12 induced *D. longicephala*, were imaged.

We used CLSM to image the body in 3D. Samples were stained with Congo Red, which binds to the crustacean exoskeleton particularly well^29^. After fixation in 3.7% formaldehyde diluted in PBS (0.1 M, pH 7.4) the samples were rinsed in PBS (0.1 M, pH 7.4) 6 times for 10 min and 2 times for 1 h. Subsequently, they were stained in Congo Red diluted in PBS (0.1 M, pH 7.4, 3 mg/ml) for 8 h and finally rinsed in PBS for 1 h shielded from light. The stained samples were transferred onto object slides prepared with ringed sticky tape^30^ as a spacer. Three ringed sticky tapes were mounted on top of each for *D. pulex*, whereas for *D. longicephala* 9 ringed sticky tapes were used. Samples were mounted in a lateral position in vectashield (Vector Laboratories, Burlingame, CA, USA) and cover-slipped. Scan depth was limited to one body hemisphere. All scans were conducted with a CLSM (Leica SP5, Leica Microsystems GmbH, Wetzlar, Germany) with an excitation wavelength of 561 nm and the emission filter set to 568 nm. Due to its size *D. longicephala* had to be scanned in multiple image stacks for each individual. These stacks were stitched before analysis, using the ImageJ plugin TrakEM2^31^.

### Carapace shape modelling and finite element analysis

To model *D. pulex* and *D. longicephala* overall shape, we applied a classical morphometric approach. In the first step, the body’s outline from a lateral view was described by a set of landmarks and semi-landmarks, using the point measurement tool of Cell^D (Olympus, Hamburg, Germany). In geometric morphometrics, landmarks are explicitly defined morphological loci e.g. the ligament insertion on a skeletal element, whereas semi-landmarks are derived from landmarks e.g. half the distance between two landmarks. We placed the origin of our coordinate system describing *D. pulex* body shape in the nauplius eye. In *D. longicephala*, we used the centre of the compound eye, as the nauplius eye is unpigmented and difficult to determine visually. We dedicated landmarks to explicit muscle-attachment points within the head region which are visible through the transparent integument, the insertion point of the tail spine and dorsal edge’s thorns as well as intermediated semi-landmarks for description of the carapace outline (Fig. 5B,C, Table 2). In the second step semi-landmarks were projected onto the carapace surface, based on the CLSM scans, using a morphologically adjusted grid. To accomplish this, a vector grid with 10 vertical and 31 horizontal lines was projected in a sagittal orientation into the image stack using Adobe Photoshop (Adobe Systems Software Ireland Limited, Dublin, Ireland). The grid was adjusted to the individual sample by bringing the vertical lines in parallel with the animal’s dorsal edge. The uppermost horizontal line was fitted to the head’s highest point, the lowermost line to the carapace–spine transition. The grid was adjusted to the animal’s body width by fitting the second vertical line to the most ventral point of the carapace and the second last vertical line to the most dorsal point of the head. The grid was coloured in white (grey-value 255) after adjusting. Then the image series including the grid was loaded into FIJI^32^ as an image stack. Contrast was corrected if necessary and the stack was superimposed via 3D Viewer^33^. In the 3D viewer, the scan was oriented laterally, the surface measurement points were collected using the multipoint tool. This was done by marking a point at every intersection of the grid, starting in the most anterior dorsal corner, leading to a surface dataset of 310 points for each individual. On each horizontal line of the grid, an additional semi-landmark was marked at the intersection with the ventral edge of the carapace. These additional semi-landmarks ensured description of the ventral cleft. In a last step, three reference points were taken at each individual for subsequent alignment of the datasets. The first point was the tip of the rostrum, second the transition of the carapace into the spine on the ventral bend, and the finally the fifth thorn on the dorsal edge, counted anteriorly from the dorsal spine. Finally, the outline data and the surface data were combined and averaged to reproduce representative *D. pulex* and *D. longicephala* shapes. Based on these combined coordinate data sets, surface models were created using the FEA software z88 Aurora (Rieg F., Universität Bayreuth, Germany) and supplied with the carapace thickness (using the shell plugin) as well as loading force and constraints. A loading force of 1 mN was applied to the nodes representing the highest lateral width of the carapace. As constraints, the nodes forming the outline of the daphniids body, except the ventral edge of the carapace, were blocked for movement in any direction to reflect the constraints of the bilateral nature of daphniids with a carapace free moving at the ventral cleft. For the material parameters of the models we used the Young’s-modulus of the procuticle determined with AFM. Poisson’s ratio was set to 0.15 and the thickness of the model was adapted to the STEM observations. Simulations were conducted for uninduced and induced animals of both species. Additionally, an induced animal with procuticle characteristics of an uninduced animal and vice versa was simulated for both species to determine the shape’s contribution to the defensive effect. All simulations were analysed for maximum deformation (node displacement after simulation) and maximum stress (stress within the model’s volume) in force per area ([Pa]).

**Table 2:** Set of outline landmarks for *D. pulex* and *D. longicephala*.

Point	Landmark definition ***D. pulex***	Landmark definition ***D. longicephala***
1	1^st^ dorsal thorn (counting in anterior direction from spine)	1^st^ dorsal thorn (counting in anterior direction from spine)
2	2^nd^ dorsal thorn	4^th^ dorsal thorn
3	3^rd^ dorsal thorn	7^th^ dorsal thorn
4	4^th^ dorsal thorn	10^th^ dorsal thorn
5	5^th^ dorsal thorn	13^th^ dorsal thorn
6	Middle distance between spine and heart lower edge (point 7)	Middle distance between spine and heart lower edge (point 7)
7	Heart lower edge, horizontally projected on the dorsal edge	Heart lower edge, horizontally projected on the dorsal edge
8	Head-carapace transition (dorsal bend)	Heart centre, horizontally projected on the dorsal edge
9	Heart upper edge, horizontally projected on the dorsal edge	Heart upper edge, horizontally projected on the dorsal edge
10	Levator lower edge, horizontally projected on the dorsal edge	Levator lower edge, horizontally projected on the dorsal edge
11	Levator upper edge, horizontally projected on the dorsal edge	Levator upper edge, horizontally projected on the dorsal edge
12	2^nd^ abductor lower edge, horizontally projected on the dorsal edge	2^nd^ abductor lower edge, horizontally projected on the dorsal edge
13	2^nd^ abductor upper edge, horizontally projected on the dorsal edge	2^nd^ abductor upper edge, horizontally projected on the dorsal edge
14	1^st^ abductor lower edge, horizontally projected on the dorsal edge	Levator upper edge, vertically projected on the head outline
15	1^st^ abductor lower edge, vertically projected on the dorsal edge	2^nd^ abductor upper edge, vertically projected on the head outline
16	1^st^ abductor upper edge, vertically projected on the head outline	Caecum dorsal edge, vertically projected on the head outline
17	Crest of the head outline	Complex eye ventral edge, vertically projected on the head outline
18	Complex eye upper edge, horizontally projected on the head outline	Complex eye lower edge, horizontally projected on the head outline (ventral)
19	Complex eye centre, horizontally projected on the head outline (ventral)	Rostrum tip
20	Complex eye lower edge, horizontally projected on the head outline (ventral)	Complex eye ventral edge, vertically projected on the rostrum edge (posterior)
21	Rostrum tip	Head-carapace transition (ventral bend)
22	Head-carapace transition (ventral bend)	Heart upper edge, horizontally projected on the ventral edge
23	Heart upper edge, horizontally projected on the ventral edge	Heart lower edge, horizontally projected on the ventral edge
24	Heart lower edge, horizontally projected on the ventral edge	Horizontal projection of point 6 on the ventral edge
25	Horizontal projection of point 6 on the ventral edge	13^th^ dorsal thorn, horizontally projected on the ventral edge
26	5^th^ dorsal thorn, horizontally projected on the ventral edge	10^th^ dorsal thorn, horizontally projected on the ventral edge
27	4^th^ dorsal thorn, horizontally projected on the ventral edge	7^th^ dorsal thorn, horizontally projected on the ventral edge
28	3^rd^ dorsal thorn, horizontally projected on the ventral edge	4^th^ dorsal thorn, horizontally projected on the ventral edge
29	2^nd^ dorsal thorn, horizontally projected on the ventral edge	1^st^ dorsal thorn, horizontally projected on the ventral edge
30	1^st^ dorsal thorn, horizontally projected on the ventral edge	Transition carapace-spine (ventral bend)
31	Carapace lower edge ventrally	Spine tip
32	Transition carapace-spine (ventral bend)	—
33	Spine tip	—

### Structural Young’s-modulus and critical force

For comparison and validation of the simulations, indentations were conducted determining the structural Young’s-modulus and critical force (the force needed to collapse the carapace resulting in lethal injury). For this purpose, *D. pulex* and *D. longicephala* were asphyxiated with acidulated water and directly transferred into a glass petri dish lubricated with Vaseline for animal fixation and filled with water. Measurements were conducted using a microindenter (Basalt I, TETRA GmbH, Illmenau, Germany) equipped with a cantilever with a spring constant of 330 N mm^−1^. For *D. pulex* a steel minutien pin was used as the indenting probe. With a tip radius of about 2.5 µm, it represented quite well the geometrical properties of the mandible tips of *C. obscuripes*. For *D. longicephala* a glass pipette was pulled to a capillary with a pipette puller (KE Pipetten Puller horizontal, H. Saur Laborbedarf, Reutlingen, Germany) and the tip was sealed over a flame. With tip radii of 12.37 µm ± 1.36 SD these resembled the size of the proboscis tip of *N. glauca*. The measurements were conducted by bringing the indenter into a position slightly above the middle of the carapace followed by indenting a predefined depth. In total 94 *D. pulex* (50 induced, 44 uninduced) and 147 *D*. *longicephala* (62 induced, 85 uninduced) were analysed for their indentation properties. Based on the obtained data the structural Young’s-modulus was determined based on the Hertz model for parabolic indenters^34, 35^ in Matlab® (MathWorks, Natick, Ma, USA):

4 $$E=\frac{4\,\sqrt{r}}{3\,}\frac{F}{1-{\vartheta }^{2}}{\delta }^{3/2}$$

*E* = effective Young’s-modulus

*F* = force

*r* = cantilever tip radius

*δ* = indentation depth

$$\vartheta $$ = Poisson’s ratio

**Equation** (4): Hertz model for parabolic tips

The critical force was defined as the force needed to collapse the carapace resulting in lethal injury to a daphniid. To evaluate the critical force rapid drops in the force–distance curves of the micro-indenter data were used to indicate structural collapse of the carapace and used to give the maximum force before collapse. During the experiment the indentation was visually monitored to guarantee that force drops were indeed collapses of the carapace.

### Statistical analysis

We used the Software STATISTICA 12 (StatSoft. Inc., Tulsa, USA) to analyse our data of the STEM observations, the AFM and microindenter measurements for statistically significant differences. Therefore, we first tested the data for normal distribution and afterwards used a suitable test to compare induced with uninduced treatments. Normally distributed data were analysed using a t-test otherwise we used a Mann Whitney U-test. For the analysis of the AFM data we used a nested design to control for inter-individual differences and afterwards tested for differences between induced and uninduced treatment.

### Data availability

The datasets generated during and analysed during the current study are available from the corresponding author on reasonable request.

## Electronic supplementary material

Supplementary Figure 1
