# Supplementary material for: Biomechanical properties of predator-induced body armour in the freshwater crustacean Daphnia
Source: Sci Rep. 2017 Aug 29;7:9750. doi: 10.1038/s41598-017-09649-5 (PMC5575280; doi:10.1038/s41598-017-09649-5)
Supplement: Supplementary file 1 — Supplementary Figure 1 [file 41598_2017_9649_MOESM1_ESM.pdf]

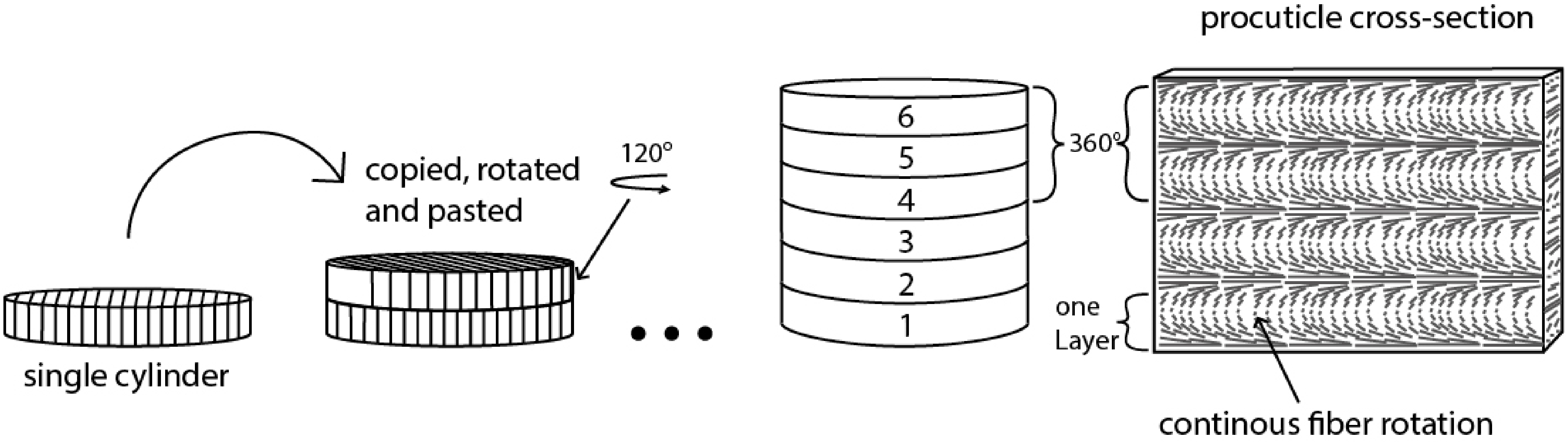

S-Figure 1: Scheme of the procuticle modelling for FEA. First a cylinder was created, subdivided into elements with alternating material properties reflecting the chitin-protein matrix, and afterwards simulated for mechanical impact. Then the initially build cylinder was copied, superimposed, rotated (120°) and pasted on top of the former cylinder, followed by the next simulation. This procedure was repeated to a maximum of 6 stacked cylinders. Each of these cylinders represented 120° of fibre rotation. Thus, three cylinders represented 360° fibre-rotation, equal to two procuticle layers, observed in STEM.

S. Kruppert, M. Horstmann, L. C. Weiss, U. Witzel, C. F. Schaber, S. N. Gorb, R. Tollrian

"Biomechanical properties of predator-induced body armour in the freshwater crustacean *Daphnia*"
